# Immune-Relevant and Antioxidant Activities of Vitellogenin and Yolk Proteins in Fish

**DOI:** 10.3390/nu7105432

**Published:** 2015-10-22

**Authors:** Chen Sun, Shicui Zhang

**Affiliations:** Laboratory for Evolution & Development, Institute of Evolution & Marine Biodiversity and Department of Marine Biology, Ocean University of China, Qingdao 266003, China; sunchen@ouc.edu.cn

**Keywords:** vitellogenin, lipovitellin, phosvitin, immunity, antioxidant activity

## Abstract

Vitellogenin (Vtg), the major egg yolk precursor protein, is traditionally thought to provide protein- and lipid-rich nutrients for developing embryos and larvae. However, the roles of Vtg as well as its derived yolk proteins lipovitellin (Lv) and phosvitin (Pv) extend beyond nutritional functions. Accumulating data have demonstrated that Vtg, Lv and Pv participate in host innate immune defense with multifaceted functions. They can all act as multivalent pattern recognition receptors capable of identifying invading microbes. Vtg and Pv can also act as immune effectors capable of killing bacteria and virus. Moreover, Vtg and Lv are shown to possess phagocytosis-promoting activity as opsonins. In addition to these immune-relevant functions, Vtg and Pv are found to have antioxidant activity, which is able to protect the host from oxidant stress. These non-nutritional functions clearly deepen our understanding of the physiological roles of the molecules, and at the same time, provide a sound basis for potential application of the molecules in human health.

## 1. Introduction

**Figure 1:**
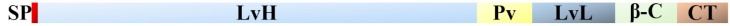
Schematic summary of structure of a complete teleost vitellogenin (Vtg). A short signal peptide (SP) is shown at the *N*-terminus, flowing by a lipovitellin heave chain (LvH), a phosphorylated serine-rich phosvitin (Pv), a lipovitellin light chain (LvL), and a β-component (β-C) plus a *C*-terminal coding region (CT).

Most fishes are oviparous, with their eggs being fertilized externally [1]. Eggs or haploid reproductive cells, which develop into viable embryos after fertilization, are the final product of oocyte growth and differentiation [2]. Generally, several steps are involved in oocyte development: formation of primordial germ-cells (PGCs), and transformation of PGCs into oogonia and then to oocytes. Subsequently, massive maternal information and molecules needed for early embryo development are deposited in growing oocytes during vitellogenesis, including RNAs, proteins, lipids, vitamins, and hormones [2,3]. One of the most important proteins deposited in oocytes is vitellogenin (Vtg), a member of the large lipid transfer protein (LLTP) superfamily [3,4,5]. Vtg is a high molecular mass glycolipophosphoprotein, usually circulating in the blood (vertebrates)/hemolymph (invertebrates) as a homodimer [4,6,7,8]. There are usually several isoforms of Vtg in a given species, which are encoded by a multigene family [9,10]. For instance, three *vtg* genes have been identified in chicken *Gallus gallus* [11,12], four in Africa frog *Xenopus laevis* [13,14], and six in nematode *Caenorhabditis elegans* [15]. Multiple *vtg* genes are also common in teleosts. It has been documented that there are seven *vtg* genes in zebrafish *Danio rerio* [16,17], two *vtg* genes in carp *Cyprinus carpio* [18], four *vtg* genes in medaka *Oryzias latipes* [10], three *vtg* genes in striped bass *Morone saxatilis* [19], and three *vtg* genes in white perch *Morone americana* [20]. All vitellogenins (Vtgs) encoded by multiple genes display a similar structure in vertebrates, such as fish, and invertebrates, particularly insects [21,22]. In most cases, Vtg contains three conserved domains, the LPD_N (also known as vitellogenin_N or LLT domain), which is identified at the *N*-terminus, the domain of unknown function (DUF) 1943, and the von Willebrand factor type D domain (vWD), which is located at the *C*-terminus and distributed over a wide range of proteins [21]. Occasionally, a domain of unknown function called DUF1944 is found to be present in between DUF1943 and vWD in some Vtg proteins from vertebrates such as chicken and fish [22]. Beginning at the *N*-terminus, a complete fish Vtg consists of a signal peptide, a lipovitellin heavy chain (LvH), a phosphorylated serine-rich phosvitin (Pv), a lipovitellin light chain (LvL), and a β-component (β-C) plus a *C*-terminal coding region (CT) comprising the vWD (Figure 1) [4,19,20,23]. Notably, some teleostean Vtgs lack Pv and much of the carboxyl-terminus (β-component and *C*-terminal peptide), consisting of only LvH and LvL [23]. Pv are also absent in most invertebrate Vtg [8,16].

Vtgs, the precursors of egg yolk proteins, are present in the females of nearly all oviparous species including fish, amphibians, reptiles, birds, most invertebrates and the platypus. Vtgs are usually synthesized in an extra-ovarian tissue (in the liver of vertebrates, the hepatopancreas of crustaceans and the fat body of insects) and transported by the circulation system to the ovary, where it is internalized into growing oocytes via receptor-mediated endocytosis during vitellogenesis with diverse proportional composition [2,7,19,24,25,26,27,28,29,30,31]. Interestingly, the rates of different Vtgs internalized by growing oocytes are not always equal to the rates of circulating Vtgs in the blood, which may be due to the regulation of the system of multiple ovarian receptors engaged in endocytosis of different Vtgs [32,33,34,35]. Once internalized into the oocytes, Vtgs are proteolytically cleaved by the aspartic protease cathepsin D to generate yolk proteins, such as Lv subunits, Pv and β-C [36,37,38,39,40,41,42,43]. Lv subunits and Pv are stored in yolk globules or platelets, while β-C remains in cytoplasm as a soluble fraction [44,45,46]. Lv, the largest yolk protein derived from the proteolytic processing of Vtgs, is an apoprotein delivering mainly phospholipids into developing oocytes [36,47]. Pv, the smallest yolk protein, largely consists of phosphorylated serine residues thought to stabilize nascent Vtg structure during lipid loading and to enhance solubility of Vtg in the blood [4,47]. β-C and CT, the small cleavage products of vWD that contains a highly conserved motif of repeated cysteine residues, are postulated to stabilize the Vtg dimer for cellular recognition and receptor binding, and to protect Vtg or its product yolk proteins from premature or inappropriate proteolysis [4,19,20]. All these yolk proteins are later used as the nutrients by developing embryos to nourish their cells [48,49].

Vtgs were once regarded as a female-specific protein [50,51]; however, synthesis, albeit in smaller quantities, has been shown to occur in male and even sexually immature animals [52,53,54], suggesting that Vtgs presumably fulfill a more general role independent of gender. Recently, both Vtgs and yolk proteins have been shown to be connected with the immune defense and antioxidant activity in fish, challenging the traditional view that Vtgs and yolk proteins were simple source of nutrients for the developing embryos. Below we will discuss the immune-relevant and antioxidant activities of Vtgs and yolk proteins in fish.

## 2. Immune-Relevant Activities of Vtgs and Yolk Proteins

### 2.1. Immune Roles of Vtgs

**Figure 2:**
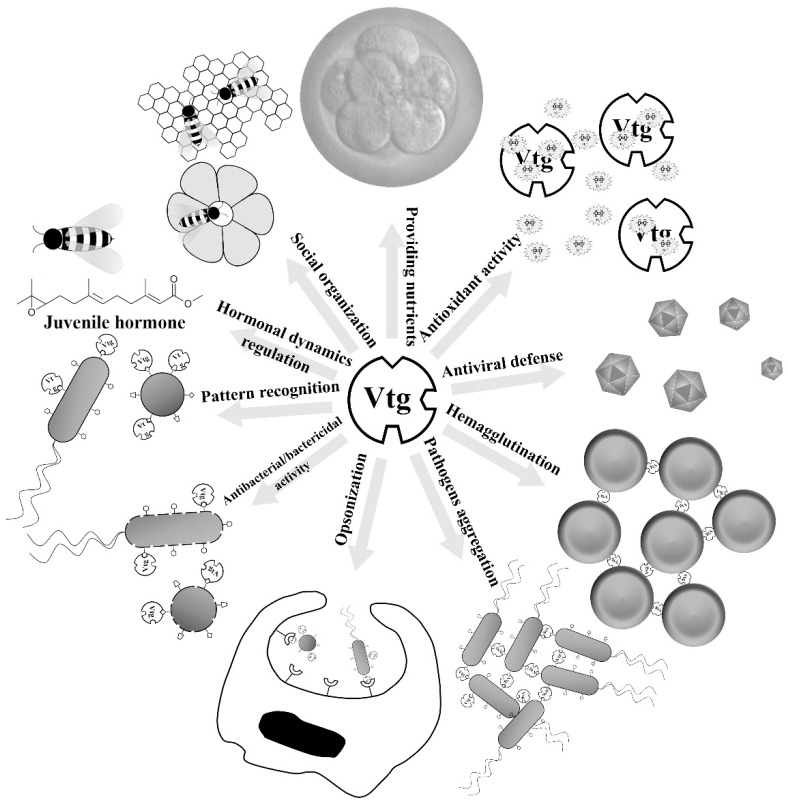
Multiple roles of vitellogenin (Vtg). Vtg is traditionally thought to provide protein- and lipid-rich nutrients for developing embryos and larvae. However, accumulating data demonstrate that its roles extend beyond the nutritional function. In the advanced eusocial insect honeybee, Vtgs were shown to be associated with the social organization, temporal division of labor and foraging specialization, regulation of hormonal dynamics and change in gustatory responsiveness. Recent studies show that Vtgs also play immune-relevant roles. Vtg is able to recognize the invading microbes as a multivalent pattern recognition receptor, kill bacteria or neutralize virus as an effector molecule as well as enhance phagocytosis as an opsonin. Besides, Vtg also exhibits activities to hemagglutinate erythrocytes and aggregate pathogens. In addition to immune roles, Vtg plays another novel role as an antioxidant.

Accumulating data demonstrated several non-nutritional roles for Vtg. For instance, Vtgs were shown to be associated with the social organization, temporal division of labor and foraging specialization, regulation of hormonal dynamics and change in gustatory responsiveness in the honeybee *Apis mellifera*, an advanced eusocial insect (Figure 2) [55,56,57,58]. Recent studies show that Vtgs also play immune-relevant roles (Figure 2). The first solid evidence showing that Vtg preforms an immune-relevant role was the observation by Zhang *et al.*, that Vtg purified from the ovaries of the protochordate amphioxus (*Branchiostoma japonicum*) exhibited hemagglutinating activity against chick, toad and grass carp erythrocytes as well as antibacterial activity against the Gram-negative bacterium *E. coli* [59]. Soon after that, Vtg purified from the rosy barb *Puntius conchonius* was found to be capable of inhibiting the growth of the Gram-negative bacteria *E. coli*, *E. aerogenes* and *Pseudomonas putida* as well as the Gram-positive bacteria *Staphylococcus aureus*, *Bacillus subtilis* and *Streptococcus pyogenes* [60], and Vtg from the carp capable of suppressing the growth of *E. coli* and *S. aureus* in a dose dependent-manner [61]. Interestingly, Vtgs from protostomes also appear to have antibacterial activity. Vtg from the scallop (*Patinopecten yessoensis*) was recently shown to have antibacterial activity against Gram-positive and Gram-negative bacteria [62]. In addition, Vtg in the nematode *C. elegans* seems also involved in its antibacterial defense. A reduced survival was observed in the *vtg*-knockdown *C. elegans* after pathogen infection [63]. Another evidence for a role of invertebrate Vtg associated with resistance against bacteria was provided by the enhancement of resistance of nematode against the pathogen *Photorhabdus luminescens*, when the production of Vtg was stimulated by estrogen 17β-estradiol and phytoestrogen daidzein. However, reduction of Vtg caused by soy isoflavone genistein diminished the host resistance to *P. luminescens* [64]. Taken together, it appears that the antibacterial activity is a universal property of Vtgs from both vertebrates and invertebrates.

Shi *et al.* showed that intraperitoneal injection of *E. coli* was able to enhance the level of serum Vtg in male *P. conchonius* [60]. This has recently been confirmed by Lu *et al.*, who showed that expression of *vtg* genes in the skin of zebrafish was induced following the challenge with Gram-negative bacterium *Citrobacter freundii* [65,66]. Moreover, an increased expression of *vtg* was also detectable in the insect *Bactericera cockerelli* after infected by “*Candidatus* Liberibacter solanacearum” via transcriptome analyses [67]. These data together suggest that Vtg may play an active role in the anti-infection of the host *in vivo*. Actually, Tong *et al.* showed that Vtg produced in male zebrafish as a consequence of induction by lipopolysaccharide (LPS) and lipoteichoic acid (LTA) is an acute phase reactant, with antibacterial activity against *E. coli* and *S. aureus* [68].

Vtg appears to play a multifaceted immune-relevant functions. Li *et al.* demonstrated that Vtg of the marine fish *Hexagrammos otakii* was able to bind to Gram-negative bacterium *E. coli* and Gram-positive bacterium *S. aureus* as well as fungus *Pichia pastoris* [69]. The binding to *E. coli* and *S. aureus* was also detected for carp and zebrafish Vtgs [61,68]. The binding of Vtgs to bacteria provides them ability to aggregate pathogens as well as to recognize the invading microbes [61]. Further examination via ELISA assay showed that Vtgs exhibited specific affinities to the components conserved within a class of microbes, called pathogen-associated molecular patterns (PAMPs), including LPS of Gram-negative bacteria, LTA of Gram-positive bacteria, peptidoglycan (PGN) of Gram-negative and positive bacteria, and glucan of fungi [61,69]. These observations indicate that Vtg first functions as a multivalent pattern recognition receptor capable of identifying invading Gram-negative and Gram-positive bacteria as well as fungi, and is involved in host immune defense as a detector. In a recent study attempting to search PGN recognition proteins in giant tiger shrimp (*Penaeus monodon*), an 83 kDa protein was isolated by *in vitro* PGN pull-down binding assay and identified as a Vtg-like protein via mass spectrometry as well as Western blots with monoclonal antibodies specific of Vtgs reported from *P. monodon* [70], implicating that invertebrate Vtg may also play a pattern recognition receptor role. Scanning electron microscopy as well as bacterial cell and protoplast lysis assays showed that *H. otakii* Vtg was able to kill pathogenic bacteria by lysing the whole cells (with cell walls) instead of protoplast (without cell walls) via interaction with LPS and LTA [71]. These show that Vtg functions as an effector molecule, capable of directly killing bacteria. Interestingly, Vtg was also shown to be able to enhance the phagocytosis of microbes by macrophages. Li *et al.* first reported that *H. otakii* Vtg could facilitate engulfing of the microbes *E. coli*, *S. aureus* and *P. pastoris* by head-kidney-derived macrophages *in vitro* [69]. Later, Vtg of carp was shown to possess similar phagocytosis-promoting activity [61]. In an *in-situ* study of impacts of urban wastewater on freshwater mussel *Elliptio complanata*, it was observed that the production of Vtg-like proteins was strongly associated with phagocytosis [72], suggesting a relation between Vtg and phagocytosis in invertebrates. Besides, *H. otakii* Vtg was found to be capable of binding to the cell surface of macrophages but not that of red blood cells [61,69]. Collectively, these observations indicate that Vtg is an opsonin functioning as a bridging molecule between host macrophages and invading pathogens, thereby leading to enhanced phagocytosis. Notably, Liu *et al.*, established that the *H. otakii* Vtg was able to opsonize the fungus *P. pastoris* for phagocytosis by macrophages isolated from sea bass *Lateolabrax japonicas*, implying that the opsonization of Vtg was not species-specific [73]. Further study revealed that Vtg-opsonized phagocytosis showed properties typical of type I phagocytosis, including pseudopod extension, tyrosine kinase dependence, and up-regulation of pro-inflammatory cytokine genes *tnf-α* and *il-1β* [73]. Therefore, Vtg is a pattern recognition receptor capable of identifying pathogens, a bactericidal molecule capable of damaging bacterial cell walls, and an opsonin capable of enhancing phagocytosis of pathogens by macrophages. The multifaceted immune-relevant activities of Vtg are in part endowed with its different domains. It was reported by Sun *et al.* that both DUF1943 and DUF1944 as well as vWD contribute to the function of Vtg as a pattern recognition receptor, and DUF1943 and DUF1944 (but not vWD) also contribute to the function of Vtg as an opsonin [21].

Recently, Garcia *et al.* showed that Atlantic salmon Vtg possessed neutralizing ability for infectious pancreatic necrosis virus [74], suggesting that Vtg is also involved in host antiviral immunity. This seems further supported by the observation that the mosquito (*Anopheles gambiae*) Vtg was able to interfere with anti-*plasmodium* response [75]. These denote that in addition to antibacterial activity, Vtg also has antiviral activity, which demands detailed study in the future.

### 2.2. Immune Roles of Yolk Proteins

Lv and Pv are the principal yolk proteins generated by the proteolytic cleavage of Vtg. As Vtg is an immune-competent molecule, it is thus reasonable to hypothesize that Lv and Pv also have similar immune activities. This hypothesis was first tested by Zhang and Zhang [76]. They demonstrated that the native Lv purified from ovulated eggs of the rosy barb *P. conchonius* was able to interact with LPS, LTA and PGN, as well as *E. coli* and *S. aureus*, but not with self-molecules such as the egg extracts prepared, indicating that Lv is a molecule capable of recognizing non-self components. Moreover, the bacterial binding activity of Lv enabled it to enhance the phagocytosis of bacteria by macrophages, suggesting that Lv is also an opsonin functional in developing embryos/larvae [76]. Similarly, Pv was also shown to play a critical role in the immunity of zebrafish embryos via acting as a pattern recognition receptor and an antimicrobial effector molecule [77]. In line with this, hen egg yolk Pv was also shown to be able to inhibit the growth of the Gram-negative bacterium *E. coli* and the Gram-positive bacterium *S. aureus* under thermal stress [78,79]. Of note, the affinity of Pv to LPS enabled the protein a capacity to neutralize endotoxin, promoting the survival rate of endotoxemia mice [79]. It was recently shown that a truncated Pv (Pt5) consisting of the *C*-terminal 55 residues of zebrafish Pv also displayed similar immune activities with Pv, including antimicrobial activity against *E. coli*, *Aeromonas hydrophila* and *S. aureus*, and specific affinity to LPS, LTA, and PGN [77]. Intraperitoneal injection of this Pv-derived peptide was able to increase the survival rate of zebrafish challenged with pathogenic *A. hydrophila* and to markedly decrease the number of the pathogen in multiple tissues, suggesting that Pt5 could inhibit multiplication/dissemination of pathogen in host as an antimicrobial agent. In addition to direct antimicrobial activity, Pt5 was also shown to be able to regulate the host immune responses via suppressing the expression of pro-inflammatory cytokine genes (*il-1β*, *il-6*, *tnf-α* and *ifn-γ*) and simultaneously enhancing the expression of anti-inflammatory cytokine genes (*il-10* and *il-4*), suggesting a dual role of Pt5 as both immune effector and modulator [80]. Recently, a mutant peptide of Pt5 (designated as Pt5e), generated by site-directed mutagenesis, was shown to have stronger bactericidal activity and LPS-neutralizing activity [81]. Besides, Sun *et al.* demonstrated that recombinant zebrafish Pv was capable of inhibiting the formation of the cytopathic effect in lymphocystis disease virus (LCDV)-infected cells and reducing the virus quantities in the infected cells as well as in the infected zebrafish, suggesting that Pv possesses an antiviral activity and participates in immune defense of host against the infection by viruses like LCDV [82]. Taken together, these data show that like Vtg, Lv and Pv are both immune-competent molecules involved in immune response of the host against invading pathogenic microbes.

## 3. Antioxidant Activities of Vtgs and Yolk Proteins

In addition to immune roles, another novel role of Vtg is antioxidant activity (Figure 2). It was first shown by Ando and Yanagida that Vtg from the eel *Anguilla japonica* was able to resist the copper-induced oxidation, and could protect the very low density lipoprotein (VLDL) against copper-induced oxidation [83]. This was the first observation reporting that Vtg has antioxidant activity, and serves to suppress the free-radical reactions in fish oocytes. Similar antioxidant activity was also suggested for the nematode (*C. elegans*) Vtg [84]. In the honeybee, Vtg was demonstrated to be able to reduce oxidative stress by scavenging free radicals, thereby increasing the lifespan in the facultatively sterile worker castes and reproductive queen castes [85,86]. The honeybee Vtg was also demonstrated in a recent study to be capable of recognizing cell damage through its binding to membrane and shielding living cells from damage by reactive oxygen species (ROS) [87]. It is clear that Vtg protects cells from ROS damage in both invertebrates and vertebrates.

It is well known that hen egg yolk Pv, as Vtg-derived major protein, show strong antioxidant activity owing to its high serine and phosphorus content, which makes this protein one of the strongest iron-chelating agents [88,89,90]. Very recently, we showed that zebrafish recombinant phosvitin (rPv) was an antioxidant agent capable of inhibiting the oxidation of the linoleic acid, and scavenging the 2,2-diphenyl-1-picrylhydrazyl (DPPH) radical. We also showed that zebrafish rPv is a cellular antioxidant capable of protecting radical-mediated oxidation of cellular biomolecules. Importantly, zebrafish rPv is non-cytotoxic to murine macrophages RAW264.7 [91]. These results show that Pv in fish is also a strong antioxidant agent. If Lv, another major Vtg-derived protein, has any antioxidant activity remains open, which is worthwhile exploring.

## 4. Potential Applications in Human Health

Antibiotics are globally utilized to control microbial infections in clinical practice up to date, however cases of resistance to the majority of antibiotic classes have been reported, which has become a serious threat to human health in many parts of the world [92,93]. It is thus essential to develop new antibiotic agents to combat these resistant pathogens. Antimicrobial proteins/peptides (AMPs) are potential candidates to solve this problem. As a protein/peptide with antimicrobial activity widely present in plants, animals and microbes, AMP commonly is a cationic and amphipathic molecule with a net positive charge and a high percentage of hydrophobic residues [94]. These structure characteristics provide AMP the ability to interact with the anionic cell wall and phospholipid membranes of microorganisms, which makes it more difficult for pathogens to evolve resistance [95]. Vtg and its derived protein Pv from oviparous species, especially teleost fishes, both display antibacterial activities with a broad antibacterial spectrum [59,60,61,62,68,71,77,78,79], and hence can be used as pro-drug to develop novel antibiotic agents. For example, based on the residual sequence of Pt5, the *C*-terminal peptide of zebrafish Pv, a total of six mutant peptides were generated by a single or double mutagenesis; among them, a mutant called Pt5e showed stronger antibacterial activities against *E. coli* and *S. aureus* [81], and was able to kill five strains of multiple drug resistance bacteria isolated from clinical cases via disturbing their cell membrane integrity (Data not shown).

Sepsis is a serious disease characterized by a systemic inflammatory response syndrome caused by infection. Severe sepsis is complicated by tissue damage and organ dysfunction, which can lead to sequential multi-organ failure followed by death [96]. The primary trigger of sepsis is thought to be LPS, or endotoxin, a major component of the cell wall of Gram-negative bacteria, which is released when bacteria grow or are abolished by antibiotics or host immunity. LPS, as a conserved molecular signature of Gram-negative bacteria, can specifically interact with Vtg, Lv, Pv and their derived peptides [21,61,62,69,71,76,77,79,81], making it possible to utilize these proteins/peptides to develop LPS-neutralizing agents for sepsis treatment. In line with this, both Pv and Pt5e exhibiting LPS-neutralizing ability had been shown to be able to promote the survival rate of endotoxemia mice. Moreover, both Pv and Pt5e displayed neither cytotoxicity to murine RAW264.7 macrophages nor hemolytic activity towards human red blood cells [79,81], suggesting that they can be a safe potential candidate for therapeutics of sepsis.

Antioxidant agents have attracted a great deal of attention in recent years because of their roles in prevention of chronic diseases and utilization as preservatives in food and cosmetics [97,98,99]. Vtg and its derived Pv both have antioxidant activities against ROS. As these proteins are components of our food source, they are thus natural antioxidant agents. These suggest that they can be can be an important antioxidant with a potential in preservation of food and cosmetics as well as in mediation of chronic disease states.

## 5. Conclusions

Vtg, the precursor of major egg yolk proteins, is traditionally thought to provide protein- and lipid-rich nutrients for developing embryos and larvae. However, accumulating data indicate that Vtg as well as its derived yolk proteins Lv and Pv also play non-nutritional functions: they are not only involved in immune defense but also antioxidant reaction. These non-nutritional functions clearly better and deepen our understanding of the physiological roles of the molecules, and at the same time, provide a sound basis for potential application of the molecules in human health.
